# The Impact of COVID-19 on Primary Educational Publishers in Australia

**DOI:** 10.1007/s12109-022-09903-3

**Published:** 2022-07-19

**Authors:** Grace Reid, Agata Mrva-Montoya

**Affiliations:** 1 Inquisitive, Sydney, Australia; 2 Department of Media and Communications, The University of Sydney, Camperdown, NSW 2006 Australia

**Keywords:** Primary educational publishing, Educational technology, COVID-19, Remote work, Digitisation

## Abstract

Based on interviews with 10 professionals from primary educational publishers and educational technology companies based in Australia, this article examines the challenges and impact of COVID-19 on publishing operations and outputs, and the future of the sector. The publishers had to deliver digital materials quickly, effectively and often for free to assist educators with the transition to remote learning, while working remotely themselves. They also had to transfers sales, support and professional learning online. Overall, while operationally challenging, the pandemic has accelerated the demand for digital products and facilitated growth of the sector.

## Introduction

Although some scholars have predicted that primary education would never be fully digital [1] they could have never foreseen the global pandemic that meant millions of students across the globe were forced to learn remotely. By mid-April 2020, 191 countries were forced to close their education institutions, impacting 1.7 billion students globally [2, 3]. This physical distance between teachers and students required a reliance on digital resources and platforms never seen before, and publishers had to respond quickly and supply the formats educators required.

COVID-19 has irreversibly impacted not only the educational landscape, but also the primary educational publishing sector which too has had to respond to challenges and disruptions caused by the pandemic. While industry publications reported broadly on the tribulations of the trade or commercial sector, little has been published about educational publishing. This paper presents the findings from 10 interviews about the impact of COVID-19 conducted in March–September 2021 with representatives from primary educational publishers and educational technology companies based in Australia.

The Australian educational publishing industry is a relatively modest and diverse sector, ranging from multinational corporations to small start-up companies. In 2021, 88 organisations producing materials for primary and educational sector were members of the Australian Publishers Association (APA). The number of APA members publishing specifically for primary schools was unknown [4]. Hargrave notes that the sector was previously “characterised by, and representative of, disruptive relationships—that is, independent Australian companies competing to survive in an industry dominated by foreign multinationals” [5]. In the twenty-first century, however, as “the creation, communication and delivery of content are cost-effective and immediate, small and emerging publishers experience a more even playing field” [6], and have been able to respond to the demand for more digital products.

This exploratory study aims to understand how these different companies responded to COVID-19, what were the key challenges, how the publishing operations and outputs changed, and what the short- and long-term consequences of the pandemic are likely to be. Following an overview of the impact of the pandemic on the various sectors of the publishing industry in Australia, the UK and the USA, we will present and discuss our findings from the interviews with publishing professionals. We will conclude with broad observations about the impact and the legacy of COVID-19, and some suggestions for further research.

## Publishing in the Times of COVID-19

Globally, the outbreak of COVID-19 has had a large impact on publishers from all sectors including academic/scholarly, educational, professional, and trade or consumer. Even though these publishers produce a variety of publications for different audiences, have distinctive publishing workflows, and use different marketing and sales models, all of them had to cancel travel and public events, and expand digital strategies to promote and distribute their output to cope with repeated lockdowns and disruptions to supply chains, and pivot to working from home.

According to the International Publisher Association’s (IPA) 2020 report *Response to Recovery,* the business of some publishers was more impacted than others, and there was typically little to no help from government agencies. Publishers were not deemed to be an essential service in most countries, despite being widely considered a driving force in “socio-cultural development and the growth of knowledge economies” [7]. The IPA assisted its members by investigating the impact of the pandemic on the industry, lobbying governments for stimulus packages and establishing the InSPIRe initiative to support recovery [8]. Not surprisingly, the IPA research shows “that markets in which publishers, booksellers, libraries, teachers, tech companies, regulators, and other publishing stakeholders came together exhibited more resilience and made faster recoveries” [9].

While publishers bemoaned the closure of in-person events, many of these were successfully moved online, with major industry fairs, such as the Frankfurt Book Fair attracting “a more diverse global representation and attendance than would otherwise be possible pre-COVID” [10]. The launch and promotion of new products have also moved online, and in the UK publishers reported using digital marketing strategies such as virtual events and social media to “promote discoverability and attract new customers” [11].

Even though publishers successfully pivoted their marketing and promotional efforts, they were affected by disruptions to the retail landscape and supply chain. In Australia, according to the industry survey carried out in July 2020, the early stages of the COVID-19 pandemic had an unequal impact on trade publishers. Large, multinational publishers reported having to delay the production of some titles, but overall did well in book sales, thanks to titles distributed via discount department stores, chains and online retailers. In contrast, independent publishers, reliant on distribution via independent bookshops, reported a significant impact of COVID-19 lockdowns on sales. As a result, some smaller publishers were eligible for the JobKeeper payments, while others introduced temporary reduction in working hours for staff [12]. The different way that larger and smaller publishers weathered the pandemic was similar in the UK [13], with publishers furloughing staff and relying on the UK government’s financial support [14].

Overall, the sales of books in Australia declined at the height of the pandemic’s first wave in 2020, but rebounded afterwards [15]. In 2021, the overall sales of books in Australia grew according to Nielsen BookScan [16]. The pandemic benefited online sales at the cost of brick-and-mortar sales, and the sales of physical books in general were also affected by the challenges of lockdowns and supply chain constraints [17]. Similarly, in both the UK and the USA book sales grew as a result of people having more leisure time in the coronavirus lockdowns [18]. In fact, the results of Nielsen Book’s survey of UK readers show that the time spent reading books almost doubled early in the pandemic [19].

While the impact of the pandemic on non-trade publishing sectors in Australia has not been previously investigated, two reports from the UK and the USA provide interesting insights into how academic, educational and scholarly publishers fared in the early months of the outbreak. Moreover, a number of articles have been published focusing specifically on journal publishing in science, technology and medicine [20–22].

The pandemic caused a surge in the pace and quantity of scientific publishing, especially in the number of articles related to COVID-19, in response to the demand for guidance in clinical practice and policy. Publishers had to manage increased manuscript submissions and publishing output, and navigate the pressure to publish fast [23]. In the trade-off between speed and accuracy, the value of output suffered. An analysis of highly disseminated COVID-19 research papers showed issues with the quality of these publications, and Khatter and colleagues argue for cautious interpretation of this research and the need for journals to balance rigour and speed to ensure high-quality publications [24].

This surge in publishing output wasn’t universal. Some scholarly publishers reported a decline in writing and publishing productivity among their authors, editors and reviewers as a result of the transition to online teaching, and the need to balance work–life pressures associated with lockdowns, home-schooling and caring duties. It is the female researchers in particular, who reported having less time to research and write [25, 26]. Beyond academia, authors and translators working in the book sector across Europe were affected by the loss of income due to “cancelled events, readings, book fairs, workshops, teaching, lectures, other public-contact-activities and residency-scholarships” as well as and the loss of sales caused by bookshops closures, delays in new releases and lack of exposure [27]. As a result, both academic and non-academic authors are faced with potential ruptures to their writing careers.

Overall, academic and scholarly publishers in the UK performed better than trade publishers due to being “less reliant on print sales and offering a largely digital portfolio” [28]. These publishers also reported a decline in print sales and an increase in digital sales. The higher digital sales did not compensate for lost print sales, though, and publishers may need to adjust their pricing models in the future, taking into account the broader impact of the pandemic on the university sector [29]. Despite the challenges, education and scholarly publishers in the UK refrained from delaying publications and focused on maintaining the production workflow and sales pipeline. They have also “initiated greater collaboration and strategic partnering during this period” [30].

In the USA, many publishers and educational technology companies responded to the pandemic by providing free resources for the K–12 market to assist students, educators and parents, with plans to convert to paid options down the track. This strategy was “an opportunity for providers to gain a place in the rapidly evolving market for educational content and services” [31]. Overall, while the textbook business was affected, educational publishers “benefitted from the shift to digital offerings” and Guren and colleagues predict that the sector will grow in line with the increase in remote and virtual learning [32].

The trend towards increased digitisation of offerings is not limited to publishing. According to the McKinsey & Company’s 2020 report, the adoption of digital products within the Asia-Pacific region has accelerated by over 10 years due to COVID-19 [33]. More than 50 percent of respondents reported “investing in technology for competitive advantage or refocusing their entire business around digital technologies” [34]. Moreover, the pandemic has also accelerated the digitisation of customer interactions. Interestingly, companies were able to respond to the challenges of COVID-19 more quickly than expected, with executives predicting it would take 454 days to implement remote working plans, when in reality it took only 10.5 days [35].

Regardless of the sector, publishers had to adjust to working from home in response to the national and local containment policies. While overall the transition has been successful, already in July 2020 publishers in the UK expressed concerns about staff productivity, the feeling of disconnection and mental wellbeing, and the possible “need to rethink office space in the future” [36]. In *The Bookseller*’s survey conducted a year later about the impact of the pandemic in the UK, many of the 78 publishing staff reported feeling “crushed, tired, unfocused and lacking in motivation”, while others enjoyed the flexible working arrangement. Overall, “around half of respondents believe that their future would see a 50–50 split between home and office” [37].

This approach is consistent with the trends in the business world globally as COVID-19 has prompted companies to continue with hybrid working environments, in which employees are able to work both remotely and in person. The 2022 IBM Center for the Business of Government’s report found that globally leading companies are favouring hybrid working conditions [38]. Employees are also favouring hybrid working conditions. A 2022 UNSW survey found that over a third of employees in Australia would like to work from home all the time, or for 80 per cent of their working week. Moreover, “almost three in five employees (59%) stated that their productivity was higher when working from home than in the office” [39]. Interestingly, in Kleinmen and Escudero’s review [40], quantitative studies point towards a negative effect of working from home on productivity during COVID-19, while qualitative studies show a more complex picture, with some workers reporting lower stress, higher efficiency, and better quality in their work. The studies show that the effects of working from home depend on “the complexity of the work performed, the need for interaction (or not) with other colleagues to complete certain tasks, the awareness of being observed and evaluated … and, even more importantly, family and workspace conditions at home”. Hence, hybrid working environments in the future will require responsive management, awareness of workers’ preferences and investments in work-from-home adaptation [41].

In addition to adopting flexible working arrangements, Brinton argues for the need for publishers in the UK to embed digital mindset, retrain and upskill print-facing staff, and pivot to digital sales, marketing, and customer service. There is a need to expand and effectively use customer data to inform business strategies. The digital capability and competencies are particularly important for academic and educational publishers [36]. In addition to developing digital skills, other key challenges according to the IPA include “promoting strong copyright frameworks, ensuring freedom to publish, contributing to global climate change, progressing diversity and inclusion” [42].

This overview has presented a complex and changing picture of how the broader publishing industry responded to COVID-19, and what the likely legacy is going to be. Against this background we are going to focus on how primary educational publishers in Australia, which operate in a specific ecosystem, have dealt with these challenges.

## Method

This research is part of a larger research project conducted as part of an Honours thesis focused on understanding of the impact of digital technologies and COVID-19 on the role of primary educational publishers [43]. Between March and September 2021, 10 semi-structured interviews with industry professionals working in primary educational publishing were carried out. The interviews were conducted and recorded via Zoom, transcribed using the Microsoft Word dictation tool and checked manually for accuracy. The transcripts were analysed using NVivo 12.

The interviews varied in lengths from 20 to 45 min and consisted of 10 questions exploring the role of the primary educational publisher, the impact of digitisation and COVID-19, and the future of the sector. This article considers the four questions that were asked in relation to COVID-19. These were:

- How has COVID-19 influenced your publishing operations and outputs?
- Did it have impact on the types of formats you produce?
- What do you think were the key challenges of COVID-19 for primary educational publishers?
- What do you think will be the lasting effects of the COVID-19 pandemic on the primary educational publishing sector?

The interview participants (eight women and two men) came from a variety of companies of different sizes and business models. Two interviewees were from small publishers, four were from medium-sized companies, three were from larger and well-established educational publishers, and one was a freelance consultant with experience working for educational publishers. Two interviewees represented companies that could be classified as EdTech, whereas the others were from or worked with traditional educational publishers. While a cohort of 10 may seem small, considering the size of the primary educational publishing industry in Australia (fewer than 88 companies), this sample is significant. It is also representative of the diversity present in the Australian primary educational publishing sector, comprising organisations that differ drastically in terms of size, history and the types of educational media produced.

## Findings

All 10 interviewees faced a range of issues because of the pandemic and the associated lockdowns, both in terms of outputs and publishing operations. As the outbreak of COVID-19 saw teachers having to deliver lessons remotely to primary school students across Australia, educational publishers were compelled to assess their offerings and see how they could best support their customers.

### Outputs

Nine out of 10 interviewees reported that they increased their digital offerings due to COVID-19. One interviewee was selling predominantly print-based products at the beginning of the pandemic, and they had to quickly digitise their resources, in particular their Foundation and Year 1 readers. They reflected that “this is not revolutionary … but it was for us”. Another interviewee similarly spoke about the need for quick turnaround to provide more digital resources to their customers. They already had an online platform so they “were set up well to do it but it’s the having to stop other work to do that work and fitting that in that was quite tricky”. Despite the pressure to quickly digitise the teaching materials, they felt it was an important undertaking to support teachers in their transition to remote teaching. Yet another participant noted that COVID-19 forced them to really “focus on digital” and be “ready in terms of that digital offering”. Previously they had taken a “staggered approach to digital”, but the pandemic put “digital” at the forefront of their minds. Ultimately, the major impact of COVID-19 on primary educational publishers was the need to produce more digital formats and deliver them fast.

Interviewees also reported producing more digital student-facing materials. One of them stated that they were already supplying “digital for teachers but not students as it just hadn’t been demanded before”. With the move to remote learning their customers wanted materials that students could complete independently. Another interviewee noted that they were able to create these products quickly and add them to their digital platform, which their user base already had access to. This is a big shift for primary educational publishing, as traditionally the majority of resources published have been teacher-led and teacher-facing.

### Sales and Support

Another area impacted by COVID-19 was publishers having to deliver their sales, professional learning and support remotely. As one individual said “we couldn’t sell anything” as their sales staff could no longer be on the ground in schools promoting their products. Another interviewee reported that “teachers didn’t have time to talk to us because they were all dealing with their own issues trying to transition to remote learning”. Sales staff had to find virtual ways to engage with their customers, which at first was challenging but then they moved to Zoom and other web-based communication platforms.

Even though sales were difficult, two interviewees explicitly reported they had great successes during the pandemic and another four noted that they saw COVID-19 as a “massive opportunity” for teachers to try their digital resources for free and see for themselves their benefits. Another one of the interviewed publishers saw “over 100,000 logins” onto their digital platform and another also reported “a massive take up” of the offer of free access. It meant that they “got a lot of goodwill and got a lot of new people exposed to [their] products”. This strategy resulted in more sales later in the year, once schools were back operating normally and they could “resume a more traditional model” of selling. By providing free access to digital resources, these publishers were able to introduce their product(s) to a larger audience, continue to develop their resources to suit their user base, and provide opportunities for their companies to capitalise on this period in the future.

While some publishers saw the offer of free digital resources to teachers as an opportunity, for others it was a dilemma. One interviewee in particular talked about how their company had to grapple with the idea of giving their products away for free and whether this would make customers undervalue their products. Their company wanted to protect their intellectual property, remain strict about how their materials were “used and reproduced” while supporting educators. They decided to provide free access to some of their reading materials and offer more support to existing customers. This was a difficult decision as there “was a lot of discussion at the time around which publishers were providing services for free and how much of their products they were giving away”. Similarly, another interviewee found that “there were a lot of expectations on primary educational publishers to support schools … by giving them your product”, which posed a quandary for businesses and their sales models.

Interestingly, the EdTech companies and large multinational companies that already had digitised resources were the ones that offered free access to their materials. In contrast, the traditional and more print-focused companies were less likely to offer free access during COVID-19, but aimed to support their customers in other ways (like digitising some of their resources).

Publishers were under great pressure to provide digital resources fast and with, at times, unrealistic expectations from their customers. One interviewee called this a “mad scramble” where publishers had to quickly react to the challenge teachers were facing and deliver digital materials. Similarly, another participant believed that “a lot of publishers were caught short in that they didn’t really have something to offer teachers when all of a sudden … [teachers] would just say what you do have in terms of offering a solution for me to teach my students remotely”. The interviewees noted that teachers put high levels of pressure on publishers to deliver these materials in those challenging and often emotionally charged times. And, as one publisher noted, “they felt this pain”.

While in general publishers struggled to maintain contact with their customers in lockdown, some interviewees reported major benefits from moving their professional learning online. Although it was a “scramble” at the beginning to organise, one interviewee stated it “was exciting in that it gave access to some people who couldn’t physically travel to a workshop before”. COVID-19 has provided new opportunities for publishers to connect and communicate with their customers from all around Australia and accelerated a wider acceptance of online professional learning. Despite their excitement for the opportunities created by online delivery, another interviewee noted that some teachers were tired and reluctant to attend webinars on Zoom as they were already burnt out from the overuse of technology in their teaching.

### Publishing Operations

The largest internal impact of COVID-19 on the publishers’ operations was the pivot to working remotely. Six interviewees found the transition to remote work fairly smooth. One participant reported that they were able to anticipate the lockdown in their state and sent employees home a week early to gather their equipment and set up their home offices. Another found that working from home improved internal communication and said that “in some ways it made it slightly easier too because people were easier to get hold of”. Another interviewee commented that the “actual job of publishing can be done from anywhere which is fantastic”.

Although working from home was a success for many of the interviewees, it was not without its challenges. Three interviewees reported feeling the loss of the collaborative environment facilitated by working in a shared physical space. One participant said that they did miss “the casual idea exchanges that often result in really good things happening”. They noted that these exchanges had severely diminished in an online environment. Another interviewee mentioned that their “editing processes were slower and less reliable”. Overall, however, the interviewees’ responses were mostly positive towards the shift to remote working.

### Legacy of COVID-19

The interviewees posited that in the post-pandemic environment teachers would value educational publishers and their support more. One interviewee thought that COVID-19 would “cement in the market’s mind and in the eye of educators … that the products, resources and services that publishers provide are more of an essential thing”, and change the attitude of some educators that educational publishers create a “lazy” teaching workforce. Another interviewee hoped that people would see the work of educational publishers as “vitally important”, particularly their digital resources, as teachers have been able to experience the wide variety and benefits of products that educational publishers can provide.

The COVID-19 pandemic also re-enforced the publishers’ key mission of supporting teachers. One interviewee stated that COVID-19 and the challenges of virtual teaching, made them “make sure that everything [they] were providing was easily accessible and clear for the kids”. They prioritised simplicity and wanted the technology to be straightforward and easily usable by all. Another interviewee found the initial period of remote learning enforced the need to support the teaching staff not only with digital resources, but also “building their technical capabilities and having really good pedagogical practices that they can do remotely”. In providing their customers with knowledge of effective online teaching strategies, they thought they could better support their customers to face the challenges of COVID-19.

The interviewees also touched upon the growing demand for more digital products. According to one publishing professional COVID-19 has “accelerated everything that we do in the digital space and everything that we do now we definitely have to have a digital offering”. The move to digital has mostly quickened an existing trend, but in the case of one publisher, this move heralded a complete change of direction as previously their company was planning to maintain print-only output. Apart from digitising their print products, they also had to digitise their internal processes and implement an ecommerce system. Interestingly, a couple of interviewees noted that although COVID-19 accelerated their move to digital products, it also highlighted the need for print resources. The educators wanted more student-facing workbooks that could be sent home, and this is something publishers will continue to develop.

Overall, the interviewed publishers noted a rise in acceptance and demand for digital technologies and resources among teachers. One interviewee stated that their customers realised that they were able to complete activities digitally and that “photo-copying and using backline masters … is not necessarily sustainable”. Another one noticed that they were now “reaching customers more easily”. The wider acceptance of digital products has the potential to positively impact publishers’ operations and sales.

The interviewees also noted the need to create more personalised and differentiated resources, as they were now seeing “children that are at all different stages of learning abilities, and as publishers [they] need to respond to that as well”. Another interviewee reported that many students struggled “in terms of their progression of learning so [publishers] have to really help teachers in terms of making sure they can respond to that shortfall and that they really help students’ progress in that area as well”.

With most of the publishers reporting they were able to transition to remote working successfully, not surprisingly all 10 of them were planning to continue with a hybrid working environment in the future. One participant noted that in the past some companies were reluctant “to let their employees work from home”, but “now with the rise of Zoom it’s going to be easier to work more flexibly and work with people that aren’t necessarily in your town or city”. One publisher reported saving a lot of money as they were no longer required to travel overseas, and instead conducted meetings virtually. The decrease of domestic and international travel is another lasting impact that the interviewees predicted would continue.

## Discussion

With much of Australia experiencing at least one lockdown in 2020–21 and with many NSW Higher School Certificate students having to sit remote trial examinations in 2021, the impact of COVID-19 on education is far-reaching and is predicted to last many years into the future. The pandemic has accelerated existing digitisation trends within the primary educational publishing sector. The interviewees reported that it has dramatically broadened the acceptance and increased the demand for digital products from educators. Such growth in the adoption of digital products was also reported in other industries according to the findings of the McKinsey & Company 2020 report.

Australian publishers were under pressure to deliver digital materials quickly, effectively and for free to assist educators with the transition to remote learning. While the provision of free access to digital materials was of concern to some of the interviewees who worried about the loss of business in the short term and the potential loss of value in the long term, others saw it as an opportunity to gain more customers and let a wider audience experience the benefits of their digital products. Similarly, Guren and colleagues [44] found that the companies in the USA who provided educational materials for free secured a place within educators’ classrooms and were able to retain many as customers once the free access to resources had ended.

Publishers not only had to provide new formats but also produce more student-facing materials and resources in digital format as a result of the COVID-19 pandemic. Digital resources had an advantage over print materials in the context of remote learning as they were easily editable and transferrable to customers. It will be interesting to see if more student-facing digital materials are produced by primary educational publishers because of COVID-19 in the future. None of the publishers interviewed have mentioned the impact of COVID on supply chain management, which plagued the trade industry. This is not surprising as the interviewees focused on the delivery of digital materials.

Publishers also needed to provide professional learning and support remotely, and digitise their sales and commerce systems in order to cope with the COVID-19 pandemic and subsequent lockdowns. This is again consistent with the increased digitisation of customer interactions noted in the McKinsey & Company 2020 report. Some publishers reported the need to expand the type of professional learning provided to include technology and best practice in online pedagogy. It will be interesting to see if the online delivery of professional learning and support continues in the future.

All interviewees had to move to remote working due to COVID-19 lockdowns, with majority finding this a positive experience. As working from home has become the new normal for primary educational publishers, the option of hybrid and/or flexible working is likely to be maintained, which again is consistent with the trends beyond the publishing industry. Interestingly, none of the interviewees mentioned the impact of COVID-19 on gender equality, be it among their authors or staff. While there were two men among the interviewees, both primary educational publishing and primary school teaching are dominated by women and perhaps the gendered experience of the pandemic wasn’t as apparent in this context. Moreover, the key challenge for publishers was to digitise the existing content, rather than to produce new resources.

The pandemic has reinforced the value of primary educational publishers and their products among educators, and helped the industry grow. The interviewees from smaller start-ups and the large multinational publishers found the outbreak of COVID-19 to be a time of great pressure but also great reward. They predicted that a wider acceptance and an increased demand for digital products will continue, as well as the need to create more personalised and differentiated resources for the students whose learning suffered during the pandemic.

## Conclusion

Overall, the COVID-19 pandemic has greatly impacted the Australian education ecosystem and consequently has had a significant impact on primary educational publishers. The publishers’ quick response to the challenges of remote learning and many companies’ decision to give away their products for free highlight the mission-driven focus of the publishers interviewed, who endeavour to support educators no matter the circumstances. Many publishers had to quickly digitise their offerings and offer more student-facing resources in digital formats. They had to digitise their operations and transfer sales, support and professional development online, all while working remotely.

The COVID-19 pandemic will no doubt leave a legacy across all industries. In the case of primary educational publishing, the sector was reminded of the need to be flexible, dynamic and focused on problem-solving. The pandemic has created a broader acceptance and a greater demand for digital products, and this is likely to continue together with a need for personalised and differentiated products to help students who have fallen behind with their learning. While operationally challenging for the interviewees, the pandemic provided opportunities for growth and reinforced the value of primary educational publishers in the minds of educators.

How lasting these changes will be, requires further research. The areas of particular interest include the development of flexible working policies within companies, the balance between print and digital resources, and the development of personalised and differentiated products. It would also be interesting to investigate how teachers perceived the support they received from publishers during the pandemic and what they think of publisher-created resources.
